# Molybdenum supply increases ^15^N-nitrate uptake by maize

**DOI:** 10.3389/fpls.2025.1546132

**Published:** 2025-04-08

**Authors:** Lílian A. Moreira, Merle Tränkner, Eduardo Mariano, Rafael Otto

**Affiliations:** ^1^ Luiz de Queiroz College of Agriculture, University of São Paulo, Department of Soil Science, Piracicaba, São Paulo, Brazil; ^2^ Institute of Applied Plant Nutrition, University of Göttingen, Göttingen, Germany; ^3^ Laboratory of Stable Isotope, Center for Nuclear Energy in Agriculture, University of São Paulo, Piracicaba, São Paulo, Brazil

**Keywords:** amino acids, molybdate, nitrate, ammonium, photosynthesis, fertilizers

## Abstract

Nitrogen (N) is widely used in maize (*Zea mays* L.) production. The supply of molybdenum (Mo) can increase the recovery of N by the plant due to the role of this micronutrient in the assimilation of nitrate through nitrate reductase (NR). We aimed to evaluate N metabolism and auxiliary measurements in maize as affected by combined N and Mo omission or supply under controlled conditions. Plants were grown for 28 d in a nutrient solution without Mo and N, with Mo and N, and under the omission of Mo or N. The treatments with omission received nutrients with foliar application or via nutrient solution after 28 d. Gas exchange, chlorophyll and anthocyanins indexes, and N accumulation were evaluated at 28, 35, and 44 d after transplanting (DAT). The amino acid profile was altered due to Mo and N supply to the plants, as well as the uptake and accumulation of nitrate. The highest biomass production was quantified in the positive control, supplied by the nutrient solution and later by the absence of Mo, being the foliar application inferior to this treatment. Maize biomass, with the omission of N and Mo, was 31 % lower than the supply of both nutrients. Molybdenum favors nitrate uptake by maize, mainly when supplied through the nutrient solution.

## 1 Introduction

Maize (*Zea mays* L.) is the main crop grown worldwide, and ≈1.16 billion Mg were produced in 2022 (FAO, 2022). This agricultural product is critical to ensuring world food security due to its several uses in human and animal nutrition (Tanumihardjo et al., 2020). In addition to these fates, maize can also be used as fuel (bioethanol), and this feedstock can reduce greenhouse gas emissions by 20 % compared to fossil fuels (Börjesson, 2009).

Despite the high production, Brazil's maize yield is lower than other top-producing countries worldwide. In the 2020/2021 growing season, maize production in the United States averaged the highest grain yield, with >11 Mg ha^-1^; in contrast, Brazil had an average of 5.0 Mg ha^-1^ (USDA, 2021). The low maize yields in the Brazilian territory result from inadequate management practices adopted in the production systems. A feasible strategy to improve maize yield levels in Brazil relies on applying fertilizers under rational rates, especially those containing nitrogen (N; Noor, 2017). Below (2008) has postulated that N is the second most crucial factor to achieve high yields, whereas climate is the first. Therefore, adequate N management is the most important and quick factor that farmers could modify to increase grain yield. Thus, in many cases, the correct supply and management of N can increase maize response to fertilizer addition, with subsequent higher grain yield (Davies et al., 2020).

Nitrogen is a nutrient generally applied to maize crops during their growing period (Yu et al., 2022). According to Duarte et al. (2003), ≈200 kg ha^-1^ of N is required to obtain or sustain high yields since the nutrient is the most extracted and exported by maize grown for grains production. Moreover, in maize and other crops, N use efficiency (NUE) can be precisely assessed by the isotope technique (i.e., isotope dilution) using ^15^N-labelled fertilizer. Understanding and improving NUE is crucial, as it ranges from 30-55 % across the globe due to the dependency of several factors, such as climate and N transformation processes in the soil, which could affect the bioavailability of N forms to plants, notably ammonium, and nitrate (Yu et al., 2022).

Many factors affecting N availability, absorption, and metabolism allow the development of strategies to increase plant N uptake and metabolism. The current technologies are focused on increasing the residence time of available N forms in soil (Cantarella et al., 2008; Sanz-Cobena et al., 2008). Nitrate and ammonium are the primary inorganic N sources in plant tissues. However, the anion must be first reduced to ammonium to be incorporated into organic molecules. The nitrate reduction (NR) is achieved by (1) nitrate reductase in the cytosol and (2) nitrite reductase in the plastids. The activity of NR is dependent on three co-factors, one of them being molybdopterin, a molybdenum (Mo)-containing co-factor.

The application of Mo in agricultural fields is not widespread to date, and some reasons can be attributed to this situation: the low dose required by the crops, which often leads producers to underestimate the importance of this nutrient, the high price per tone of the nutrient, and the difficulty of distributing this nutrient in the field (Oliveira et al., 2022). Moreover, only a few studies have reported its effect on the physiological, biochemical, and agronomical parameters of crops (Calonego et al., 2010; Zoz et al., 2012). Most of these studies have been carried out in legume crops due to the role of Mo in the nitrogenase enzyme, which is responsible for biological N fixation (Mengel and Kirkby, 1987). However, it is well known that this nutrient directly affects the plant N metabolism since Mo is a constituent of one of the prosthetic groups (MoCo) of several enzymes that are essential to N and C metabolism, such as nitrate reductase, aldehyde oxidase, xanthine dehydrogenase, sulfite reductase and mARC (Campbell, 1999; Zimmer and Mendel, 1999; Mendel and Haensch, 2002; Zdunek-Zastocka and Lips, 2003; Havemeyer et al., 2006). Therefore, due to the great interference of Mo in N metabolism, plants with Mo deficiency usually show aspects of low N availability as a visual symptom, and the addition of such micronutrient in crops may promote physiological and biochemical alleviation, which may result in greater root development and better N utilization with regards to biomass production (Wei et al., 2007; Škarpa et al., 2013).

Most of the studies carried out with Mo have been focused on the comparison among fertilizer sources and application methods, even though it is still not clear how these practices interfere with nitrate uptake via roots (Wei et al., 2007; Calonego et al., 2010; de Albuquerque et al., 2012; Zoz et al., 2012; Sapucay et al., 2016; Silva et al., 2017). As a result of these investigations, researchers reported that N metabolism was unbalanced (Moussa et al., 2022). The absence of Mo leads to higher nitrate content in plant tissues, as this nutrient is a constituent of nitrate reductase. Plant response to Mo deficiency is the accumulation of nitrate in the cell, but it is uncertain whether this accumulation can reduce the uptake of this N form by the plant (Hu et al., 2002; Yu et al., 2022). We hypothesized that the presence of Mo in the solution increases nitrate uptake and reduces its accumulation in plant tissues. Therefore, the objectives were to (1) evaluate the N forms taken up by maize in the presence and absence of Mo associated with N levels; and (2) provide a better understanding of the relationship between Mo supply and N metabolism, which can contribute for improving N use efficiency towards a more sustainable agriculture.

## 2 Material and methods

### 2.1 Growth conditions and experimental design

Maize seedlings (Likeit variety) were treated with a 10% sodium hypochlorite solution for 3 min, then placed on the germination paper moistened with a 1mM CaSO_4_·2H_2_O solution for 7 d. Two plants were transplanted to nutrient solution in 5-L pots with 25% ionic strength and omission or presence of Mo and N and left for 3 d. The nutrient solution was changed, and plants were exposed to a half-strong solution (50% ionic strength) for an additional 3 d. Subsequently, the plants received a nutrient solution with 100% ionic strength for 28 d. This solution had the following composition: 2 mM K^15^NO_3_ (0.775 ^15^N atom %), 0.25 mM (NH_4_)_2_HPO_4_, 0.75 mM Ca(NO_3_)_2_·4H_2_O, 0.125 mM CaCl_2_·2H_2_O, 1 mM CaSO_4_·2H_2_O, 1 mM MgSO_4_·7H_2_O, 0.025 mM Ca(H_2_PO_4_)_2_·H_2_O, 0.3 mM Fe-EDTA, 3 µM H_3_BO_3_, 2.5 µM MnSO_4_·H_2_O, 1 µM ZnSO_4_·7H_2_O, 1 µM CuSO_4_·5H_2_O and 0.3 µM Na_2_MoO_4_.2H_2_O. Therefore, the nitric pool had an abundance of 0.60 ^15^N atom %, while the ammoniacal pool presented a ^15^N natural abundance (0,366 ^15^N atom %). Unlabeled dicyandiamide (DCD) at 7 μmol L^-1^ was added as a nitrification inhibitor to suppress the conversion of NH_4_^+^ to NO_3_^−^ (Song et al., 2011). The nitrification inhibition capacity by DCD was verified in a pre-test, and its efficiency was assured for up to 4 d, representing the changeover time for solutions. Nutrient solutions were constantly aerated, and the pH was adjusted daily to 5.9 by adding CaCO_3_.

A glasshouse experiment was conducted for 44 d with 14 h of light and 10 h of dark. The experiment consisted of a completely randomized design using six treatments and four replications, totaling 24 experimental units. The treatments were: +Mo+N, positive control (complete nutrient solution); -Mo-N, negative control (low N; 5 % of N complete nutrient solution, and without Mo all the time); -Mo+N_R_ (N_R_: N resupplying in solution after 28 d growth); -Mo_R_+N (Mo_R_: Mo resupplying in solution after 28 d growth); -Mo_L_+N (-Mo_L_: Mo resupplying in leaf after 28 d growth); and -Mo+N (without Mo all the time). The foliar application was carried out only once, providing each plant with 6.7 mg of Mo. The volume of the solution regulated the amount of Mo applied, which had a concentration of 7.0 mmol L^-1^ of Na_2_MoO_4_·2H_2_O. A total of 10 mL of this solution was used for each plant and at the time of application. During the application, the surface of the pots was protected until the solution dried on the plants. The Mo in the nutrient solution was supplied continuously.

**Figure 1:**
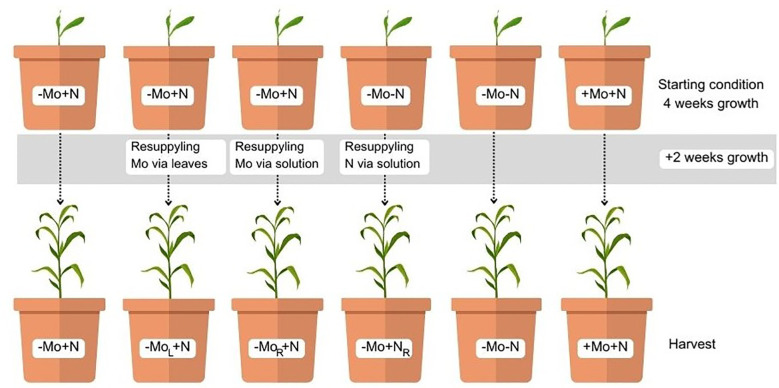
Treatment scheme. +Mo+N, -Mo-N, and -Mo+N solutions at 28 DAT. Subsequently, plants in the -Mo+N condition were subdivided into -Mo+N, -Mo_L_+N, and -Mo_R_+N, while the -Mo-N treatment was divided into -Mo-N and -Mo+N_R_. R: nutrient added through the nutrient solution; L: nutrient added through the leaves.

The plants were grown for 6 d in lower strength solutions for adaptation, after which the solution was added at 100% of the strength. In the following 22 d, the plants were exposed to the +Mo+N, -Mo-N, -Mo+N solutions; at 28 DAT, the plants in the -Mo+N condition were subdivided into -Mo+N, -MoR+N, -MoL+N and the -Mo-N treatment was divided into -Mo-N and -Mo+NR to determine whether the late supply of nutrients would be able to reverse the nutritional stress (**Figure 1**).

### 2.2 Plant measurements

At 28, 35, and 44 DAT, the youngest fully expanded leaf (leaf+1; first leaf from the apex with visible dewlap) was used to measure plant chlorophyll (*i.e.*, measurement of leaf “greenness”) and anthocyanins indexes (*i.e.*, an estimate of antioxidant pigments that may protect plants from excessive absorption of visible light) indexes with a leafclip optical sensor (Dualex, Pessl Instruments, Weiz, Austria). The same leaves were subsequently used to quantify gas exchange parameters with a portable photosynthesis system (GFS-3000, Heinz Walz GmbH, Pfullingen, Germany) between 9:00 am and 4:00 pm. The following conditions were used: chamber size 4 × 1 cm^2^, 23 °C air temperature, 55% relative humidity, CO_2_ concentration set to 400 ppm, and photosynthetic active radiation (PAR) of 1000 µmol m^–2^ s^–1^ of photons provided by LED lamps. After stabilization, measurements of the CO_2_ assimilation rate (*A*), stomatal conductance (*gs*), internal CO_2_ concentration in the substomatal chamber (*Ci*), evapotranspiration (*E*), and instantaneous water-use efficiency (WUE) were performed for 10 min in each pot.

Afterward, the central part of the leaf+1 (excluding the midrib) and a subsample of the root system (primarily the root tip zone) were sampled to assess total amino acids (at 28 and 35 DAT) and N derived from nitric- and ammoniacal-fertilizers (Ndff; at 28, 35, and 44 DAT). Both plant fractions were washed with deionized water and oven-dried at 65°C until constant weight. The plant tissues were ground in a Wiley mill to pass through a 0.5 mm sieve. To quantify the concentration of amino acids, dried plant material was hydrolyzed with 6 M HCl at 100°C for 24 h (Fountoulakis and Lahm, 1998). Then, samples were vacuum-dried with NaOH for amino acid measurements. The depicted results were obtained by ultra-high-performance liquid chromatographic-diode array (UHPLC-DAD, Agilent 1290 Infinity II LC, Agilent Technologies, Santa Clara, CA, USA) after the derivatization of amino groups. The total N concentration and ^15^N abundance in leaves and roots were determined in an elemental analyzer (ANCA-GSL, Sercon Ltd., Crewe, UK) coupled to an isotope ratio mass spectrometer (EA-IRMS; Hydra 20/20, Sercon Ltd., Crewe, UK) by weighting a subsample (≈6 mg) of maize dry biomass into a Sn capsule. Atmospheric N_2_ (0.3663 ^15^N atom %) represented the international standard, while (NH_4_)_2_SO_4_ with 1.0 ^15^N atom % was used as reference material for anchoring. The N derived from nitric fertilizers [K^15^NO_3_ and Ca(NO_3_)_2_·4H_2_O; Ndff_NF_] in maize leaves and roots was calculated according to Trivelin et al. (1994) as follows:


(1) $${\text{Ndff}}_{\text{NF}}(\%)=(\text{a}/\text{b})\times 100$$

where *a* and *b* are the ^15^N abundance (^15^N atom % in excess) in the plant and nitric fertilizers, respectively.

After non-destructive measurements at 44 DAT, the plants in each pot were removed and partitioned into leaves, stalk, and roots. The plant tissues were processed (washing, drying, grinding, etc.) as previously described. Subsequently, a subsample of each plant compartment was processed and analyzed for total N concentration and ^15^N abundance in the EA-IRMS above. The leaves and stalk compartments were pooled to represent the maize shoots. In addition to the measurement of total N accumulation by maize, the Ndff in each compartment (leaves, stalk, and roots) for the nitric (using Equations 1–5) and ammoniacal fertilizers was also calculated as below:


(2) $${\text{NA}}_{\text{plant}}({\text{mg pot}}^{-1})=\text{BP}\times \text{NC}$$

(3) $${\text{Ndff}}_{\text{NF}}({\text{mg pot}}^{-1})={\text{Nddf}}_{\text{NF}}(\%)/100\times {\text{NA}}_{\text{plant}}$$

(4) $${\text{Ndff}}_{\text{AF}}({\text{mg pot}}^{-1})={\text{NA}}_{\text{plant}}–{\text{Ndff}}_{\text{NF}}({\text{mg pot}}^{-1})–{\text{NA}}_{\text{seed}}$$

(5) $${\text{Ndff}}_{\text{AF}}(\%)={\text{Nddf}}_{\text{AF}}({\text{mg pot}}^{-1})/{\text{NA}}_{\text{plant}}\times 100$$

where *NA_plant_* is the N accumulation in maize (mg plot^-1^); *BP* is the dry biomass production (g pot^-1^); and *NC* is the total N concentration in plant tissues (mg g^-1^); Ndff_AF_ (mg pot^-1^) is the amount of N derived from ammoniacal fertilizer; and *NA_seed_* is the N accumulation in two maize seeds (mg pot^-1^).

For determination of Mo in plant tissues, 300 mg of dry biomass were microwave digested in 3 mL concentrated HNO_3_ and 2 mL of 30 % H_2_O_2_ at 200°C and 15 bar for 120 min (Tränkner et al., 2016), and the concentration of such micronutrient was measured with an inductively coupled plasma mass spectrometer (ICP-MS), followed by Mo accumulation in maize compartments.

### 2.3 Statistical analysis

Experimental data were analyzed using R (version 3.3.6, R Core Team, 2023) with RStudio as an integrated desktop environment (version 2022.07.1 + 554, Posit Software, Boston, MA, USA). Treatments were subject to one-way ANOVA; in case of significance, means were separated using Duncan test at *p* ≤ 0.05. Multivariate analysis was carried out on the leaf amino acids, and the differences between the vectors of treatment means were verified using the canonical variable analysis (CAN) method in SAS (version 9.4M3, Sas Institute Inc., Cary, NC, USA). The scores of the canonical variables were separated using Scheffé’s test (*p* ≤ 0.05).

## 3 Results

### 3.1 Pigments and leaf gas exchange parameters

**Figure 2:**
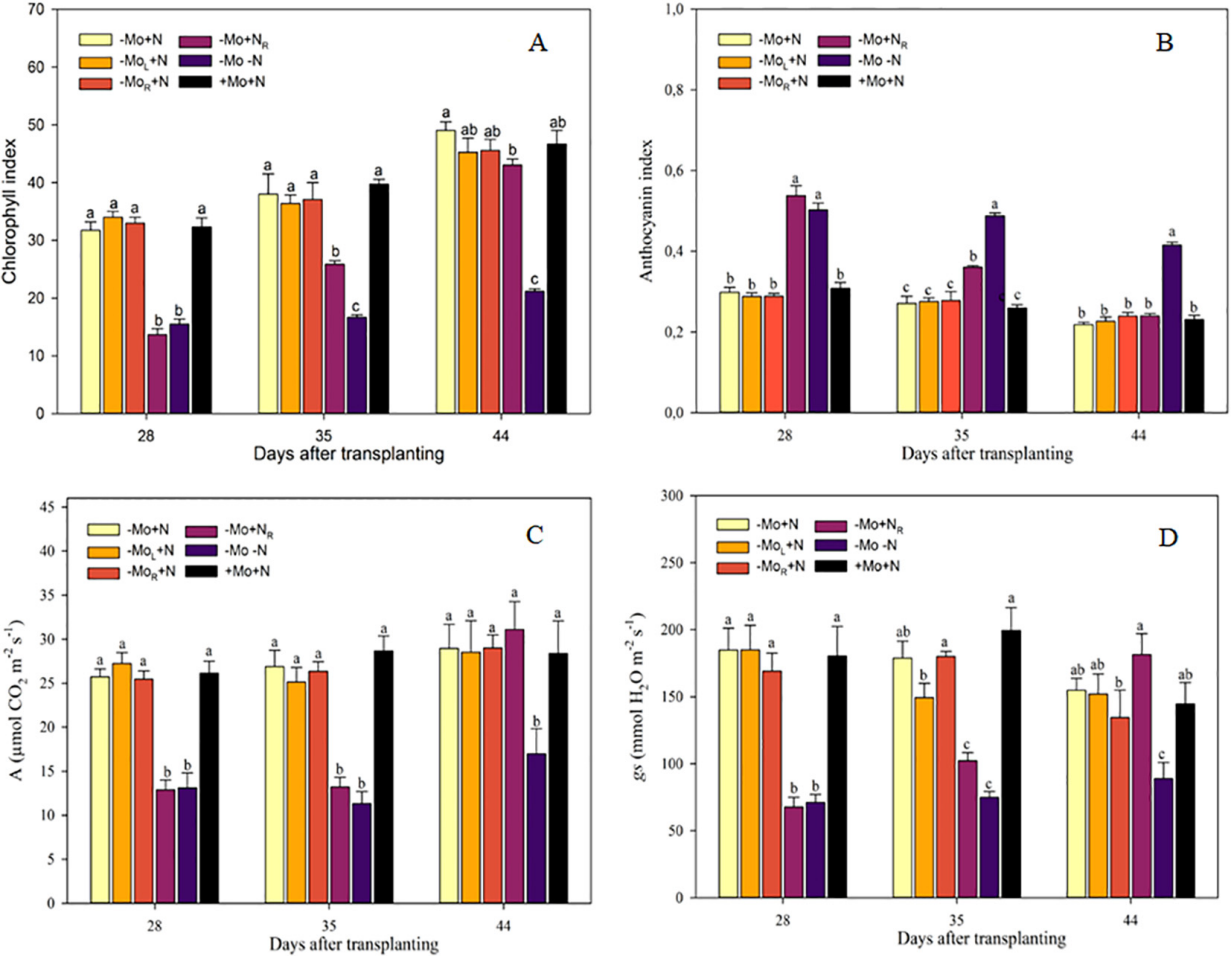
Chlorophyll index **(A)**, anthocyanin index **(B)**, CO_2_ assimilation rate (*A*; **C**), and stomatal conductance (*gs*; **D**) in maize plants grown on nutrient omission up to 28 DAT and afterward with resupply. -Mo+N, without Mo; -Mo_L_+N, Mo supply through the leaves after 28 DAT; -Mo_R_+N, Mo supply through nutrient solution after 28 DAT; -Mo+N_R_, N supply through the nutrient solution after 28 DAT; -Mo-N, negative control; +Mo+N, positive control. Vertical bars indicate the standard error of the mean. Means followed by a common letter are not significantly different according to the Duncan test (*p ≤* 0.05).

The chlorophyll index was influenced by the -Mo-N and -Mo+N_R_ treatments throughout the crop cycle (**Figure 2A**). The absence of these nutrients represents a reduction of 47 % in the chlorophyll index in comparison to the other treatments with continuous N supply at 28 DAT. Conversely, the subsequent N supply (-Mo+N_R_) increased the chlorophyll index by 45% at 35 DAT compared to -Mo-N (negative control). At 16 d after N introduction (44 DAT), the chlorophyll index of -Mo+N_R_ was equal to +Mo+N (positive control). The opposite was observed with the anthocyanin index, where -Mo-N and -Mo+N_R_ treatments increased by 76 % compared to the other treatments at 28 DAT (**Figure 2B**). The subsequent N supply (-Mo+N_R_) decreased anthocyanin production over time, exhibiting values similar to those of the treatments with continuous N at 44 DAT. Gas exchange parameters presented the same pattern as the chlorophyll index, with increasing values of *A* and *gs* over time following N introduction through the nutrient solution (**Figures 2C, D**).

### 3.2 Amino acids concentration

In the root, only alanine (Ala), asparagine (Asp), glutamine (Glu), glycine (Gly), leucine (Leu), and valine (Val) were quantified since the rest were below the detection limit (BDL; **Supplementary Table 1**). Overall, the complete N omission or even the introduction of such nutrient led to the lowest concentrations of amino acids, notably Asp, Glu, and Leu at 28 DAT. At 35 DAT, Asp, Glu, Gly, Leu, and Val concentrations were BDL in the -Mo-N treatment. The Mo omission did not affect the amino acids profile overall, except for Val, where +Mo+N (positive control) was the lowest, similar to the treatment with N addition through the nutrient solution (-Mo+N_R_).

**Figure 3:**
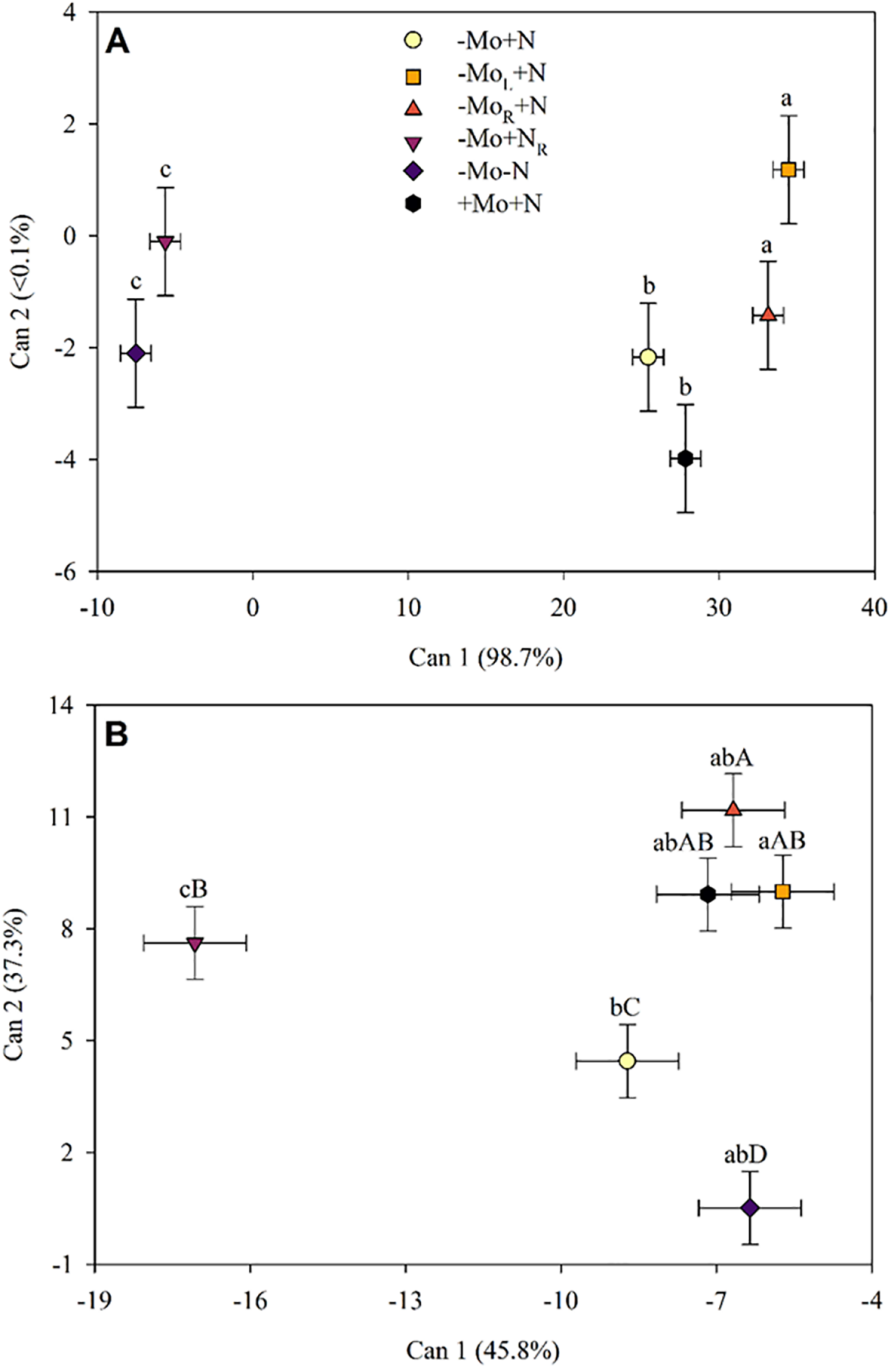
Canonical variables for the amino acids profile in maize leaves at 28 **(A)**, and 35 DAT **(B)**. -Mo+N, without Mo; -Mo_L_+N, Mo supply through the leaves after 28 DAT; -Mo_R_+N, Mo supply through the nutrient solution after 28 DAT; -Mo+N_R_, N supply through the nutrient solution after 28 DAT; -Mo-N, negative control; +Mo+N, positive control. Bi-directional bars indicate the 95% confidence interval. Treatments followed by a lowercase (CAN1) or uppercase (CAN2) common letter are not significantly different according to the Sheffé test (*p*<0.05).

The canonical variables (Can1) indicated that the lowest concentration of amino acids in maize leaves occurred when N was omitted at 28 DAT (**Figure 3A**). Plants with N but without Mo supply showed higher levels of free amino acids, except the -Mo+N treatment, where the amount of free amino acids in the plant was the same as the positive control treatment (+Mo+N). Following the Mo and N supply (35 DAT), the plants recovered (**Figure 3B**). The concentrations of free amino acids in the plant when Mo was added to the solution or through the leaves were similar to the +Mo+N. Thus, it was possible to observe the formation of three groups after nutrient supply, namely: the first consisted of the treatments with Mo supply (root and leaf) and the +Mo+N; the second consisted of the negative control (-Mo-N) and absence of Mo (-Mo+N); and the third group with N supply in the solution.

### 3.3 Biomass production and nitrogen and molybdenum accumulation

The biomass production was influenced by N and Mo supply (**Table 1**). In the roots, Mo supply via solution increased biomass production by 19 % compared to the micronutrient omission (-Mo+N) or foliar application (Mo_L_+N). Furthermore, Mo supply was equivalent to +Mo+N treatment. Nitrogen supply at 28 DAT did not recover root development. The omission of both nutrients caused a lower rate of root development, with the most significant effect attributed to N (**Supplementary Tables 2, 3**). A similar pattern was observed for the shoots (**Table 1**). However, Mo omission or supply by solution did not differ from the +Mo+N treatment. The highest biomass production considering the entire plant was observed for -Mo+N and +Mo+N (**Table 1**), while the Mo supply via foliar application was lower, followed by the treatments with N resupply or omission, which were the lowest.

**Table 1:** Dry biomass production in roots, shoots (stalk plus leaf), and entire plant of maize grown at 44 d after transplanting (DAT) on Mo and N omission and resupply.

Treatment	Roots (g pot^-1^)	Shoots (g pot^-1^)	Entire plant (g pot^-1^)
-Mo +N	37.3 ± 2.5 bc	166.7 ± 12.5 a	204.4 ± 8.9 a
-Mo_L_ +N	39.4 ± 1.6 b	141.5 ± 6.9 b	180.9 ± 5.1 b
-Mo_R_ +N	47.4 ± 2.7 a	166.7 ± 2.6 a	214.1 ± 2.2 a
-Mo +N_R_	28.2 ± 2.7 d	91.9 ± 1.8 c	120.1 ± 2.3 c
-Mo -N	30.6 ± 0.5 cd	92.4 ± 4.5 c	123.1 ± 4.0 c
+Mo +N	44.2 ± 3.2 ab	172.3 ± 5.1 a	216.6 ± 4.0 a
*p*-value	<0.010	<0.010	<0.010

Mean ± standard error of the mean (*n* = 4).

-Mo+N, without Mo; -Mo_L_+N, Mo supply in leaves after 28 DAT; -Mo_R_+N, Mo supply in roots after 28 DAT; -Mo+N_R_, N supply in roots after 28 DAT; -Mo-N, control negative; +Mo+N, control positive. Letters in the same column indicate a difference between treatments by Duncan test (p<0.05). Average followed by the standard error of the mean.

Molybdenum accumulation in maize altered upon its addition (continuous supply or subsequent addition through the leaves or nutrient solution) and exposition time to the micronutrient (**Table 2**). In the roots, Mo was only detected in the +Mo+N and -Mo_R_+N treatments; the other treatments had values BDL (1 mg kg^-1^) of the ICP-MS (**Supplementary Table 2**). The Mo accumulation in the roots under Mo resupply through nutrient solution was 60% lower than the +Mo+N treatment. In the shoots, the Mo accumulation was much higher (17-fold higher, on average) in the treatment with Mo addition through the leaves than with Mo resupply via roots or continuous Mo supply. A similar trend was found for the entire maize plant, with high Mo accumulation when the micronutrient was added through foliar spray compared to nutrient solution. The highest N accumulation in the roots was observed in the -Mo_L_+N, -Mo_R_+N, and +Mo+N, while -Mo+N_R_ and -Mo-N were the lowest (**Table 3**). In the shoots, a similar pattern was verified. In the entire plant, the N omission throughout the crop cycle resulted in maize plants with the lowest biomass production, while N resupply increased biomass in comparison to the negative control but persisted lower than those treatments under continuous N.

**Table 2:** Molybdenum accumulation in roots, shoots (stalk plus leaf), and entire plant of maize grown at 44 d after transplanting (DAT) on Mo and N omission and resupply.

Treatment	Roots (mg pot^-1^)	Shoots (mg pot^-1^)	Entire plant (mg pot^-1^)
-Mo +N	BDL	BDL	BDL
-Mo_L_ +N	BDL	2.67 ± 1.19 a	2.67 ± 0.40 a
-Mo_R_ +N	0.17 ± 0.07 b	0.18 ± 0.08 b	0.35 ± 1.19 b
-Mo +N_R_	BDL	BDL	BDL
-Mo -N	BDL	BDL	BDL
+Mo +N	0.42 ± 0.18 a	0.13 ± 0.06 b	0.56 ± 0.25 b
*p*-value	<0.01	<0.01	<0.01

Mean ± standard error of the mean (*n* = 4).

BDL, below detection limit; -Mo+N, without Mo; -Mo_L_+N, Mo supply in leaves after 28 DAT; -Mo_R_+N, Mo supply in roots after 28 DAT; -Mo+N_R_, N supply in roots after 28 DAT; -Mo-N, control negative; +Mo+N, control positive. Letters in the same column indicate a difference between treatments by Duncan test (*p*<0.05). Average followed by the standard error of the mean.

**Table 3:** Nitrogen accumulation in roots, shoots (stalk plus leaf), and entire plant of maize grown at 44 d after transplanting (DAT) on Mo and N omission and resupply.

Treatment	Roots (g pot^-1^)	Shoots (g pot^-1^)	Entire plant (g pot^-1^)
-Mo +N	0.80 ± 0.06 ab	3.85 ± 0.21 a	4.65 ± 0.23 ab
-Mo_L_ +N	0.87 ± 0.04 a	3.73 ± 0.13 ab	4.60 ± 0.11 ab
-Mo_R_ +N	0.96 ± 0.05 a	3.44 ± 0.18 b	4.41 ± 0.22 b
-Mo +N_R_	0.62 ± 0.04 bc	2.54 ± 0.12 c	3.16 ± 0.15 c
-Mo -N	0.44 ± 0.02 c	1.12 ± 0.05 d	1.56 ± 0.05 d
+Mo +N	0.99 ± 0.10 a	3.98 ± 0.08 a	4.97 ± 0.13 a
*p*-value	<0.01	<0.01	<0.01

Mean ± standard error of the mean (*n* = 4).

-Mo+N, without Mo; -Mo_L_+N, Mo supply in leaves after 28 DAT; -Mo_R_+N, Mo supply in roots after 28 DAT; -Mo+N_R_, N supply in roots after 28 DAT; -Mo-N, control negative; +Mo+N, control positive. Letters in the same column indicate a difference between treatments by Duncan test (*p*<0.05). Average followed by the standard error of the mean.

**Figure 4:**
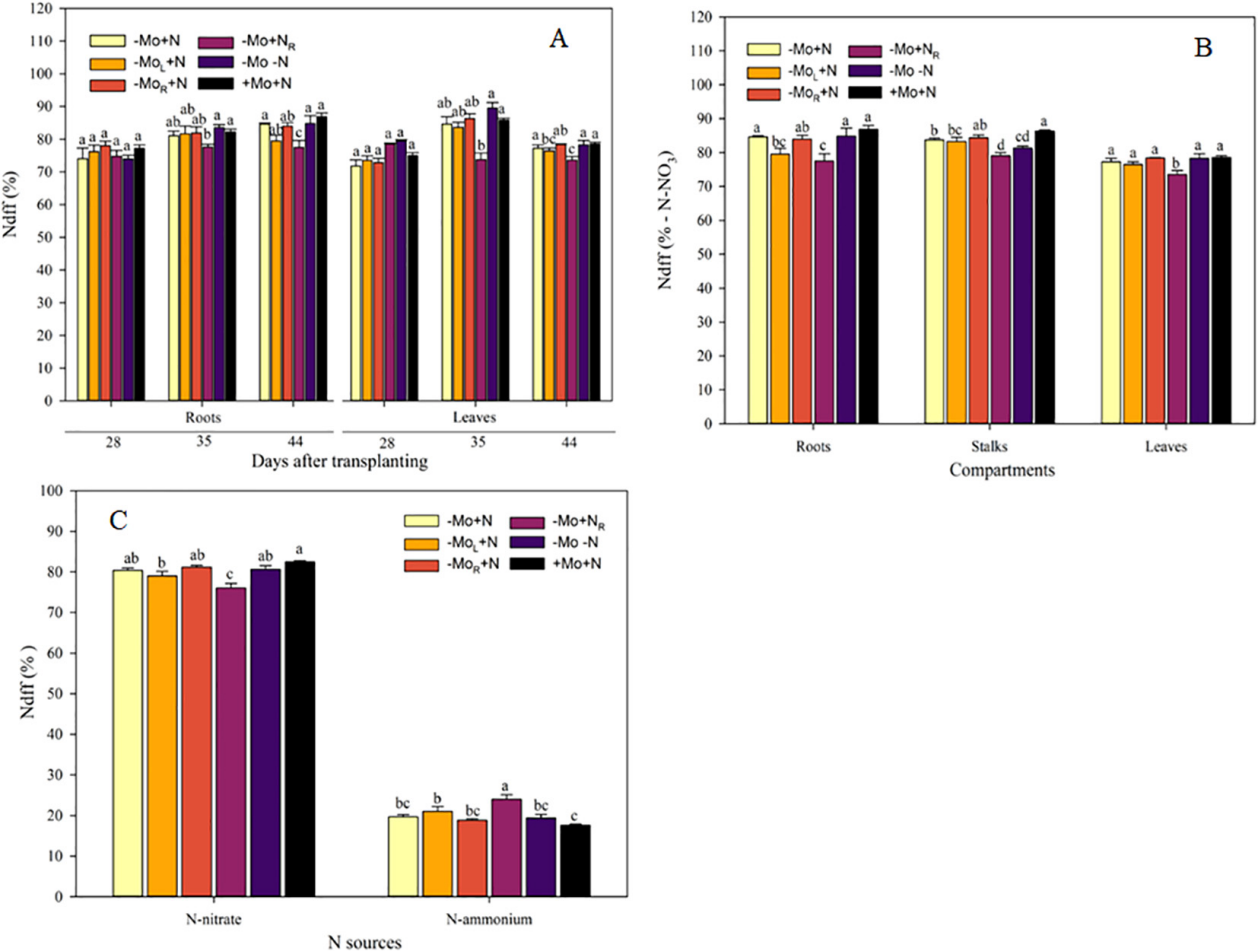
Nitrogen from nitric fertilizer during the experiment **(A)**, in the compartments **(B)** and whole plant **(C)**. -Mo+N, without Mo; -Mo_L_+N, Mo supply through the leaves after 28 DAT; -Mo_R_+N, Mo supply through the nutrient solution after 28 DAT; -Mo+N_R_, N supply through the nutrient solution after 28 DAT; -Mo-N, negative control; +Mo+N, positive control. Vertical bars indicate the standard error of the mean. Means followed by a common letter are not significantly different according to the Duncan test (*p ≤* 0.05).

Regarding Ndff_NF_ in the roots, at 28 DAT, the omission of Mo and N did not alter the nitric-N contribution (**Figure 4A**). However, at 35 DAT, it was observed that the addition of N without Mo supply decreased the Ndff_NF_ by 5.2% compared to +Mo+N. In the leaves, the -Mo+N_R_ treatment had the lowest NO_3_^-^-N uptake at 35 and 44 DAT, although the Mo addition through the leaves (-Mo_L_+N) was similar to this treatment in the last sampling time. The treatments influenced Ndff_NF_ across the plant compartments at 44DAT (**Figure 4B**). In the roots, Ndff_NF_ was lower in -Mo_L_+N and -Mo+N_R_ treatments were lower than the -Mo+N, -Mo-N, and +Mo+N. In the stalks, the Mo supply through the nutrient solution was similar to the positive control (+Mo+N). In the leaves, the N resupply led to the lowest nitric-N contribution (Ndff_NF_) to the plant metabolism. Lastly, the Ndff_NF_ and Ndff_AF_ were affected by the Mo and N supply (**Figure 4C**). The maize +Mo+N treatment was higher than the -Mo_L_+N and -Mo+N_R_ relative to the contribution of nitric-N to maize. On the other hand, the ammoniacal-N had the highest contribution under N resupply (-Mo+N_R_), while treatments with continuous N were overall similar.

## 4 Discussion

The chlorophyll index in maize was higher in the presence of N, a finding supported by the literature. At least 50 % of the N in leaves is found in the chloroplasts, where chlorophyll is the pigment responsible for “greenness” (Onoda et al., 2017). Furthermore, under nutritional stress conditions, the plant will decrease chlorophyll content and increase the production of other pigments for photoprotection. The supply of N reduced the anthocyanins production and increased the production of chlorophyll, which was not observed for Mo, where in general the changes in pigments were imperceptible. Hadi et al. (2016) determining plant pigments observed that the Mo addition increased them, but this condition may be associated with the presence of cadmium in a contaminated soil. This helps to understand gas exchange parameters, where plants with N omission presented lower values of *A* and *gs*. Molybdenum decreased *gs* compared to the resupply of N, and the Mo supply resembled the *gs* to the control, indicating ease in plant adaptation to regulate stomatal conductance in the presence of Mo (Schwarz and Mendel, 2006). These results are also reported for wheat plants under stressful conditions, Mo helps in the abscisic acid production and interferes with stomatal control (Sun et al., 2009; Wu et al., 2014). In other words, here we see that even with an adequate nutrients supply, low Mo levels hinder the plant's osmotic control.

The biomass production responded to Mo supply through the nutrient solution (-Mo_R_+N), yielding the same as the +Mo+N, while application via foliar spray decreased maize biomass. Based on the amino acid profile and Mo accumulation values in maize, the application of this micronutrient foliar was toxic to the plant. Excess Mo can reduce plant photosynthetic rates, reduce the amount of chlorophyll and biomass, and impair Cu uptake, resulting in reduced lignification (Xu et al., 2018). It is important to emphasize that the damage caused by excess Mo is rarely reported in the literature and it is often not possible to identify visual symptoms in plants, as occurred in this study. Therefore, the foliar rate used was inadequate to supply the nutrient, resulting in physiological damage. Kovács et al. (2015) observed a slightly lower biomass production regardless of the Mo rate in the nutrient solution compared to the Mo-unfertilized treatment, where the latter exhibited lower leaf contents, in agreement with our findings, because a lower availability of N, compared to nutrient solution and foliar application, resulted in better production. The lack of response to Mo can be explained by the seed quality, which, if grown in soils with a high Mo content, will not respond to fertilization due to the plant's low demand for the nutrient.

In most of the available literature, the nitrate accumulation in plants is lower in leaves under Mo supply compared to those without supply of this micronutrient (Calonego et al., 2010; Kovács et al., 2015; Santos et al., 2018). In our study, the Mo application favored nitrate uptake from nitric fertilizers in the hydroponic solution and their utilization by plants. Kovács et al. (2015) demonstrated that maize seedlings had lower nitrate accumulation in the leaves and increased its content in the roots due to the rate of ammonium molybdate supplied through hydroponics. The lower presence of free nitrate-N in the leaves is frequently reported when Mo is applied, regardless of the crop evaluated (Sun et al., 2009; Xiong et al., 2001; Ide et al., 2011), suggesting the reduction of this mineral N by the nitrate reductase, which is a crucial enzyme to convert nitrate to ammonium and ultimately to organic N forms. In addition to this role, Mo also acts in the metabolism of sulfur and is consequently related to protein production. It also participates in abscisic acid synthesis and makes up the mARC enzyme, which is linked to nitric oxide production in plants, which can also be formed by the increased expression of nitrate reductase due to the accumulation of nitrite in the cells (Imran et al., 2019). Wu et al. (2020) related that enhancing photosynthesis by Mo increases water use efficiency, leading to greater tolerance of abiotic stresses. Nitrogen accumulation in the plant was higher when both nutrients (N and Mo) were available, and Mo omission slightly compromised N accumulation. We observed that Mo and N resupply did not recover N accumulation in the plant. The period of N resupply in the plants grown in omission was after the V4 phase (i.e., four leaves fully expanded), which may have favored the impairment of the development of these plants. Molybdenum is constantly omitted in Poaceae, and here we demonstrate that its application contributes to the adequate development of plants because it favors cellular metabolism and the production of metabolites involved in plant protection, stomata regulation, and N utilization.

## 5 Conclusions

Molybdenum favors nitrate uptake by maize plants, primarily when supplied in the nutrient solution. The Mo omission rapidly changed the stomatal conductance, evidencing the importance of this nutrient in plant osmoregulation, which was confirmed by the amino acids and their distribution. The biochemical processes triggered by the Mo presence that result in increased nitrate uptake need further research to be explained, as it is probably not just nitrate reductase activity that interferes in this process.

## Data Availability

The raw data supporting the conclusions of this article will be made available by the authors, without undue reservation.

## References

[B1] Below F. E. (2008). “The seven Wonders of the corn yield world,” in 2008 Illinois Crop Protection Technology Conference, vol. 2008. (Urbana-Champaign University of Ilinois at Urbana-Campaign). Available at: https://cropphysiology.cropsci.illinois.edu/seven-wonders-of-the-corn-yield-world/ (Accessed June 15, 2015).

[B2] Börjesson P. (2009). Good or bad bioethanol from a greenhouse gas perspective – What determines this? Appl. Energy. 5, 589–594. doi: 10.1016/j.apenergy.2008.11.025

[B3] Calonego J. C. Junior E. U. R. Barbosa R. D. Leite G. H. P. Grassi Filho H. (2010). Nitrogen topdressing fertilization on common bean with leaf spray of molybdenum. Rev. Ciênc. Agron. 3, 334–340. doi: 10.1590/S1806-66902010000300003

[B4] Campbell W. H. (1999). Nitrate reductase structure, function and regulation: Bridging the Gap between. Annu. Rev. Plant Biol. 1, 277–303. doi: 10.1146/annurev.arplant.50.1.277 15012211

[B5] Cantarella H. Trivelin P. C. O. Contin T. L. M. Dias F. L. F. Rossetto R. Marcelino R.. (2008). Ammonia volatilisation from urease inhibitor-treated urea applied to sugarcane trash blankets. Sci. Agric. 4, 397–401. doi: 10.1590/S0103-90162008000400011

[B6] Davies B. Coulter J. A. Pagliari P. H. (2020). Timing and rate of nitrogen fertilization influence maize yield and nitrogen use efficiency. PLoS One 5), e0233674. doi: 10.1371/journal.pone.0233674 PMC7259653 32469984

[B7] de Albuquerque H. C. Pegoraro R. F. Vieira N. M. B. De Jesus Ferreira Amorim I. Kondo M. K. (2012). Nodulor capability and agronomic characteristics of common bean plants subjected to fragmented molybdenum and nitrogen fertilization. Rev. Ciênc. Agron. 2, 214–221. doi: 10.1590/S1806-66902012000200002

[B8] Duarte A. P. Kiehl J. C. Camargo M. A. F. Reco P. C. (2003). Accumulation of dry matter and nutrients in tropical and temperate maize cultivars introduced in Brazil. RBMS 3, 1–20. doi: 10.18512/1980-6477/rbms.v2n3p1-20

[B9] Food and Agriculture Organization of the United Nations. (2022). FAOSTAT. Available online at: http://www.fao.org/faostat/en/data/QC (Accessed February 14, 2024).

[B10] Fountoulakis M. Lahm H.-W. (1998). Hydrolyzsis and amino acid composition analysis of proteins. J. Chromatogr A. 826, 109–134. doi: 10.1016/S0021-9673(98)00721-3 9917165

[B11] Hadi F. Ali N. Fuller M. P. (2016). Molybdenum (Mo) increases endogenous phenolics, proline and photosynthetic pigments and the phytoremediation potential of the industrially important plant *Ricinus communis* L. for removal of cadmium from contaminated soil. Environ. Sci. pollut. Res. 23, 20408–20430. doi: 10.1007/s11356-016-7230-z 27457556

[B12] Havemeyer A. Bittner F. Wollers S. Mendel R. Kunze T. Clement B. (2006). Identification of the missing component in the mitochondrial benzamidoxime prodrug-converting system as a novel molybdenum enzyme. J. Biol. Chem. 281, 34796–34802. doi: 10.1074/jbc.M607697200 16973608

[B13] Hu C. Wang Y. Wei W. (2002). Effect of molybdenum applications on concentrations of free amino acids in winter wheat at different growth stages. J. Plant Nutr. 25, 1487–1499. doi: 10.1081/PLN-120005404

[B14] Ide Y. Kusano M. Oikawa A. Fukushima A. Tomatsu H. Saito K.. (2011). Effects of molybdenum deficiency and defects in molybdate transporter MOT1 on transcript accumulation and nitrogen/sulphur metabolism in Arabidopsis thaliana. J. Exp. Bot. 62, 1483–1497. doi: 10.1093/jxb/erq345 21131548

[B15] Imran M. Hu C. Hussain S. Rana M. S. Riaz M. Afzal J.. (2019). Molybdenum-induced effects on photosynthetic efficacy of winter wheat (*Triticum aestivum* L.) under different nitrogen sources are associated with nitrogen assimilation. Plant Physiol. Biochem. 141, 154–163. doi: 10.1016/j.plaphy.2019.05.024 31163342

[B16] Kovács B. Puskás-Preszner A. Huzsvai L. Lévai L. Bódi É. (2015). Effect of molybdenum treatment on molybdenum concentration and nitrate reduction in maize seedlings. Plant Physiol. Biochem. 96, 38–44. doi: 10.1016/j.plaphy.2015.07.013 26226599

[B17] Mendel R. R. Haensch R. (2002). Molybdoenzymes and molybdenum cofactor in plants. J. Exp. Bot. 53, 1689–1698. doi: 10.1093/jxb/erf038 12147719

[B18] Mengel K. Kirkby E. A. (1987). Principles of plant nutrition. 4. ed (Bern: International Potash Institute).

[B19] Moussa M. G. Sun X. El-Tohory S. Mohamed A. Saleem M. H. Riaz M.. (2022). Molybdenum role in nitrogen bioavailability of wheat-soil system using the natural ^15^N abundance technique. J. Soil Sci. Plant Nutr. 3, 3611–3624. doi: 10.1007/s42729-022-00913-w

[B20] Noor M. A. (2017). Nitrogen management and regulation for optimum NUE in maize – A mini review. Cogent Food Agric. 1, 1348214. doi: 10.1080/23311932.2017.1348214

[B21] Oliveira S. L. Crusiol C. A. C. Rodrigues V. A. Galeriani T. M. Portugual J. R. Bossolani J. W.. (2022). Molybdenum foliar fertilization improves photosynthetic metabolism and grain yields of field-grown soybean and maize. Front. Plant Sci. 13, 887682. doi: 10.3389/fpls.2022.887682 35720532 PMC9199428

[B22] Onoda Y. Wright I. J. Evans J. R. Hikosaka K. Kitajima K. Niinemets Ü.. (2017). Physiological and structural tradeoffs underlying the leaf economics spectrum. New Phytol. 4, 1447–1463. doi: 10.1111/nph.2017.214.issue-4 28295374

[B23] R Core Team (2023). R: A Language and Environment for Statistical Computing (Vienna, Austria: R Foundation for Statistical Computing). Available at: https://www.R-project.org/ (Accessed January 26, 2024).

[B24] Santos R. L. D. Freire F. J. Oliveira E. C. A. D. Barbosa J. D. A. Moura M. J. A. D. Lopes N. R. D. C.. (2018). Sampling of sugarcane leaves in field experiments to determine the activity of nitrate reductase. Commun. Soil Sci. Plant Anal. 1, 76–87. doi: 10.1080/00103624.2017.1421648

[B25] Sanz-Cobena A. Misselbrook T. H. Arce A. Mingot J. I. Diez J. A. Vallejo A. (2008). An inhibitor of urease activity effectively reduces ammonia emissions from soil treated with urea under Mediterranean conditions. Agric. Ecosyst. Environ. 4, 243–249. doi: 10.1016/j.agee.2008.02.001

[B26] Sapucay M. J. L. Da C. Vieira R. F. Carneiro J. E. De S. Paula Júnior T. J. D. De Lima M. S. Vieira R. F.. (2016). Is it possible to attain high-yielding common bean using molybdenum fertilizer instead of side-dressed nitrogen? J. Plant Nutr. 11, 1644–1653. doi: 10.1080/01904167.2016.1161782

[B27] Schwarz G. Mendel R. R. (2006). Molybdenum cofactor biosynthesis and molybdenum enzymes. Ann. Rev. Plant Biol. 57, 623–647. doi: 10.1146/annurev.arplant.57.032905.105437 16669776

[B28] Silva A. Franzini V. I. Piccolla C. D. Muraoka T. (2017). Molybdenum supply and biological fixation of nitrogen by two Brazilian common bean cultivars. Rev. Bras. Eng. Agric. Ambient. 2, 100–105. doi: 10.1590/1807-1929/agriambi.v21n2p100-105

[B29] Škarpa P. Kunzová E. Zukalová H. (2013). Foliar fertilization with molybdenum in sunflower (Helianthus annuus L.). PSE 4, 156–161. doi: 10.17221/663/2012-PSE

[B30] Song W. Makeen K. Wang D. Zhang C. Xu Y. Zhao H.. (2011). Nitrate supply affects root growth differentially in two rice cultivars differing in nitrogen use efficiency. Plant Soil 1, 357–368. doi: 10.1007/s11104-011-0723-0

[B31] Sun X. Hu C. Tan Q. Liu J. Liu H. (2009). Effects of molybdenum on expression of cold-responsive genes in abscisic acid (ABA)-dependent and ABA-independent pathways in winter wheat under low-temperature stress. Ann. Bot. 2, 345–356. doi: 10.1093/aob/mcp133 PMC2710908 19491090

[B32] Tanumihardjo S. A. McCulley L. Roh R. Lopez-Ridaura S. Palacios-Rojas N. Gunaratna N. S. (2020). Maize agro-food systems to ensure food and nutrition security in reference to the Sustainable Development Goals. Glob. Food Secur. 25, 100327. doi: 10.1016/j.gfs.2019.100327

[B33] Tränkner M. Jàkli B. Tavakol E. Dittert K. Senbayram M. (2016). Magnesium deficiency decreases biomass water-use efficiency and increases leaf water-use efficiency, and oxidative stress in barley plants. Plant Soil. 406, 409–423. doi: 10.1007/s11104-016-2886-1

[B34] Trivelin P. C. O. Lara Cabezas W. A. R. Victoria R. L. Reichardt K. (1994). Evaluation of a ^15^N plot design for estimating plant recovery of fertilizer nitrogen applied to sugar cane. Sci. Agric. 51, 226–234. doi: 10.1590/S0103-90161994000200005

[B35] Unites States Department of Agriculture (2021). Foreign Agricultural Service- USDA (World Agriculture Production). Available at: https://apps.fas.usda.gov/psdonline/circulars/production (Accessed July 20, 2021).

[B36] Wei L. P. Li Y. R. Yang L. T. (2007). Effects of molybdenum on nitrogen metabolism of sugarcane. Sugar Tech. 1, 36–42. doi: 10.1007/BF02956911

[B37] Wu S. Hu C. Tan Q. Nie Z. Sun X. (2014). Effects of molybdenum on water utilization, antioxidative defense system and osmotic-adjustment ability in winter wheat (Triticum aestivum) under drought stress. Plant Physiol. Biochem. 83, 365–374. doi: 10.1016/j.plaphy.2014.08.022 25221925

[B38] Wu S. Hu C. Yang X. Tan Q. Yao S. Zhou Y.. (2020). Molybdenum induces alterations in the glycerolipidome that confer drought tolerance in wheat. J. Exp. Bot. 71, 5074–5086. doi: 10.1093/jxb/eraa215 32369576

[B39] Xiong L. Ishitani M. Lee H. Zhu J. K. (2001). The Arabidopsis LOS5/ABA3 locus encodes a molybdenum cofactor sulfurase and modulates cold stress- and osmotic stress-responsive gene expression. Plant Cell. 13, 2063–2083. doi: 10.1105/tpc.010101 11549764 PMC139452

[B40] Xu S. Hu C. Tan Q. Qin S. Sun X. (2018). Subcellular distribution of molybdenum, ultrastructural and antioxidative responses in soybean seedlings under excess molybdenum stress. PPB 123, 75–80. doi: 10.1016/j.plaphy.2017.11.023 29223849

[B41] Yu X. Keitel C. Zhang Y. Wangeci A. N. Dijkstra F. A. (2022). Mo favors nitrate uptake by maize plants and its late supply can recover the utilization of nitrate, mainly by root uptake. Agric. Ecosyst. Environ. 338, 108089. doi: 10.1016/j.agee.2022.108089

[B42] Zdunek-Zastocka E. Lips H. S. (2003). Plant molybdoenzymes and their response to stress. Acta Physiol. Plant 4, 437–452. doi: 10.1007/s11738-003-0026-z

[B43] Zimmer W. Mendel R. (1999). Molybdenum metabolism in plants. Plant Biol. 1, 160–168. doi: 10.1111/j.1438-8677.1999.tb00239.x

[B44] Zoz T. Steiner F. Testa J. V. P. Seidel E. P. Fey R. Castagnara D. D.. (2012). Foliar fertilization with molybdenum in wheat. Ciênc. Agr. 2, 633–638. doi: 10.5433/1679-0359.2012v33n2p633

