# Supplementary material for: Molybdenum supply increases 15N-nitrate uptake by maize
Source: Front Plant Sci. 2025 Apr 8;16:1546132. doi: 10.3389/fpls.2025.1546132 (PMC12011875; doi:10.3389/fpls.2025.1546132)
Supplement: Supplementary file 1 [file DataSheet1.docx]

**Table 1.** Amino acid concentration in maize roots grown at 28 and 35 d after transplanting (DAT) on Mo and N omission nutrient resupply (35 DAT). Mean ± standard error of the mean (*n* = 4).

| **Treatment** | **-Mo +N** | **-Mo_L_ +N** | **-Mo_R_ +N** | **-Mo +N_R_** | **-Mo -N** | **+Mo +N** |
| --- | --- | --- | --- | --- | --- | --- |
| **28 DAT** | | | | | | |
| **Ala** | 91.5±25.6 abc | 78.5±12.0 bc | 116.8±17.0 ab | 51.8±3.3 c | 59.7±4.8 bc | 143.0±23.7 a |
| **Asp** | 66.0±7.4 a | 57.5±5.3 a | 76.3±5.3 a | 13.3±11.5 b | 11.8±10.2 b | 76.5±8.5 a |
| **Glu** | 74.0±8.6 a | 63.5±6.2 a | 87.3±7.8 a | 13.3±11.5 b | 13.0±11.3 b | 92.3±10.6 a |
| **Gly** | 68.3±9.6 a | 56.8±5.0 a | 73.3±7.9 a | BDL | BDL | 73.5±8.8 a |
| **Leu** | 59.7±7.5 a | 55.5±4.8 a | 66.5±5.2 a | 11.3±9.7 b | BDL | 63.0±6.4 a |
| **35 DAT** | | | | | | |
| **Ala** | 106.8±27.4 abc | 119.25±8.8 ab | 155.8±14.0 a | 58.8±4.2 cd | 45.0±1.5 d | 80.0±13.7 bcd |
| **Asp** | 57.0±17.0 a | 65.8±5.4 a | 58.0±2.2 a | 47.7±4.9 a | BDL | 58.8±7.7 a |
| **Glu** | 63.3±19.7 a | 70.5±6.8 a | 72.3±6.6 a | 47.1±5.0 a | BDL | 61.3±8.9 a |
| **Gly** | 56.8±18.5 a | 52.0±15.2 a | 43.0±12.9 a | 45.4±4.5 a | BDL | 63.3±6.5 a |
| **Leu** | 60.3±4.3 a | 58.5±0.6 a | 47.0±0.6 a | 34.1±6.9 b | BDL | 55.8±4.0 a |
| **Val** | 51.9±4.6 a | 52.2±0.5 a | 42.8±0.7 a | 11.3±9.7 b | BDL | 14.0±12.1 b |

BDL: below the detection limit. -Mo+N: without Mo; -Mo_L_+N: Mo supply through the leaves after 28 DAT; -Mo_R_+N: Mo supply through the nutrient solution after 28 DAT; -Mo+N_R_: N supply through the nutrient solution after 28 DAT; -Mo-N: negative control; and +Mo+N: positive control. Means followed by a common letter in the row are not significantly different according to the Duncan test (*p*≤0.05).

**Table 2.** Molybdenum concentration in roots, stalk and leaf plant of maize grown at 44 d after transplanting (DAT) on Mo and N omission and resupply. Mean ± standard error of the mean (*n* = 4).

| **Treatment** | **Roots (mg kg^-1^)** | **Stalk (mg kg^-1^)** | **Leaf (mg kg^-1^)** |
| --- | --- | --- | --- |
| **-Mo +N** | BDL | BDL | 1.33± b |
| **-Mo_L_ +N** | BDL | 1.40±0.62 a | 33.75±15.09 a |
| **-Mo_R_ +N** | 3.75± 1.67 b | BDL | 2.20±0.98 b |
| **-Mo +N_R_** | BDL | BDL | 2.63±1.17 b |
| **-Mo -N** | BDL | BDL | BDL |
| **+Mo +N** | 9.58±4.28 a | 0.28±0.12 b | 11.75±5.25 b |
| ***p*-value** | <0.01 | <0.01 | <0.01 |

BDL: below detection limit, -Mo+N: without Mo, -Mo_L_+N: Mo supply in leaves after 28 DAT, -Mo_R_+N: Mo supply in roots after 28 DAT, -Mo+N_R_: N supply in roots after 28 DAT, -Mo-N: control negative, and +Mo+N: control positive. Letters in the same column indicate a difference between treatments by Duncan test (*p*<0.05). Average followed by the standard error of the mean.

**Table 3.** Nitrogen concentration in roots, stalk and leaf plant of maize grown at 44 d after transplanting (DAT) on Mo and N omission and resupply. Mean ± standard error of the mean (*n* = 4).

| **Treatment** | **Roots (dag kg^-1^)** | **Stalk (dag kg^-1^)** | **Leaf (dag kg^-1^)** |
| --- | --- | --- | --- |
| **-Mo +N** | 2.13±0.95 a | 1.56±0.70 c | 3.22±1.44 ab |
| **-Mo_L_ +N** | 2.23±1.00 a | 2.20±0.98 b | 3.28±1.47 a |
| **-Mo_R_ +N** | 2.13±0.95 a | 1.55±0.69 c | 2.87±1.28 ab |
| **-Mo +N_R_** | 2.19±0.98 a | 2.79±1.24 a | 2.77±1.23 b |
| **-Mo -N** | 1.46±0.65 b | 0.91±0.41 d | 1.38±0.61 c |
| **+Mo +N** | 2.36±1.04 a | 1.67±0.75 c | 3.10±1.38 ab |
| ***p*-value** | <0.01 | <0.01 | <0.01 |

-Mo+N: without Mo, -Mo_L_+N: Mo supply in leaves after 28 DAT, -Mo_R_+N: Mo supply in roots after 28 DAT, -Mo+N_R_: N supply in roots after 28 DAT, -Mo-N: control negative, and +Mo+N: control positive. Letters in the same column indicate a difference between treatments by Duncan test (*p*<0.05). Average followed by the standard error of the mean.
